# Globalization and Loss of Plant Knowledge: Challenging the Paradigm

**DOI:** 10.1371/journal.pone.0037643

**Published:** 2012-05-25

**Authors:** Ina Vandebroek, Michael J. Balick

**Affiliations:** Institute of Economic Botany, The New York Botanical Garden, Bronx, New York, United States of America; University of Arkansas, United States of America

## Abstract

The erosion of cultural knowledge and traditions as a result of globalization and migration is a commonly reported phenomenon. We compared one type of cultural knowledge about medicinal plants (number of plants reported to treat thirty common health conditions) among Dominican laypersons who self-medicate with plants and live in rural or urban areas of the Dominican Republic (DR), and those who have moved to New York City (NYC). Many plants used as medicines were popular Dominican food plants. These plants were reported significantly more often by Dominicans living in NYC as compared to the DR, and this knowledge was not age-dependent. These results contradict the popular paradigm about loss of cultural plant knowledge and is the first study to report a statistically measurable increase in this type of knowledge associated with migration.

## Introduction

The loss of biodiversity-based cultural knowledge is widely reported, globally as well as at the level of communities and individuals, despite the importance of plants for human health and subsistence in developing and developed nations [1]–[9]. Loss of biological resources, an increasingly globalized society, cultural homogenization and desire for modernization are all factors attributed to the general decline in cultural knowledge about plants, and the disappearance of traditional practices involving these plants [6], [9]. Medicinal plant knowledge has been identified as particularly vulnerable to loss worldwide [5], [10], among others due to increasing reliance on biomedical healthcare, devaluation of the occupation of traditional herbal practitioner by younger generations, lack of cultural support and a push by some governmental programs to “modernize” medical practice [6], [11]–[14], but see also [15].

International migration, the temporary or permanent settlement of people originating from one country in another, is an important aspect of globalization [16]. In the case of migration to metropolitan areas such as London or New York, changes in knowledge about traditional uses of medicinal plants can be particularly pronounced [17]–[20]. In these new (usually multiethnic) environments, migrants are confronted with a variety of societal, economic and environmental pressures [19], [21], as well as with a shift in the prevalence of health conditions as compared to their home countries [19]. There are also constraints on the availability of plant species formerly used as botanical remedies for traditional healthcare in their home countries [18], [21]. These factors can contribute to loss of knowledge about particular plant remedies. At the same time, a culturally diverse metropolis offers the opportunity for new residents to experiment with, and to integrate knowledge and practices from other cultures, thereby making it possible to expand the original knowledge base developed in their home countries [21], [22].

Urban ethnobotany, the study of plants used by people in urban environments, is a rapidly developing field [23], [24]. Applied to the context of migration, urban ethnobotany offers the opportunity to evaluate ethnobotanical data within a larger, transnational framework by comparing plant knowledge and use by the same cultural group in its original and new environment. Here, we investigate the transnational dynamics of immigrants' medicinal plant knowledge from their origin in the Dominican Republic (DR) to their new home environment in New York City (NYC). Dominicans are the second largest Latino community in NYC after Puerto Ricans and over the past decade increased at approximately twice the rate of the city's overall Latino population [25]. Study participants were queried systematically on their knowledge of plants reported to treat thirty common health conditions. We hypothesized that Dominicans who migrated to NYC would report on average significantly less medicinal plants to treat these conditions than Dominicans who were living in the DR, because in NYC there will be more healthcare options, less availability of culturally familiar medicinal plants, and higher pressures of acculturation and modernization.

## Results

### Medicinal plants: Food versus nonfood medicines

During the survey, it was observed that Dominicans often reported food plants used as medicines (hereafter named food medicines). These are plants primarily used for consumption as foods and culinary purposes in Dominican culture that are also used secondarily for medicinal purposes. In NYC, they make up 39% of the total plant inventory of 300 plants. The mean number of food medicines a person reported ± s.d. was 13.7±5.8 for NYC (N = 165) and 12.1±5.7 for DR (N = 128). The most frequently mentioned food medicines in NYC were lime and lemon (limón, *Citrus limon* (L.) Osbeck and *Citrus aurantiifolia* (Christm.) Swingle), bitter orange (naranja agria, *Citrus aurantium* L.), cinnamon (canela, *Cinnamomum verum* J.Presl and *Cinnamomum* spp.), garlic (ajo, *Allium sativum* L.) and coconut (coco, *Cocos nucifera* L.). In comparison, plant species without any food-related uses in Dominican culture (and hence only used as medicines) are considered here nonfood medicines. The mean number of nonfood medicines reported ± s.d. was 8.9±5.3 for NYC and 12.8±8.0 for DR. The most frequently mentioned nonfood medicines in NYC were aloe (sábila, *Aloe vera* (L.) Burm.f.), castor bean (higuereta, *Ricinus communis* L.), lemongrass (limoncillo, *Cymbopogon citratus* Stapf), Guinea hen weed (anamú, *Petiveria alliacea* L.) and chamomile (manzanilla, *Matricaria recutita* L.). Lemongrass was categorized as a nonfood medicine because no culinary uses were found associated with this species in Dominican culture.

**Figure 1:**
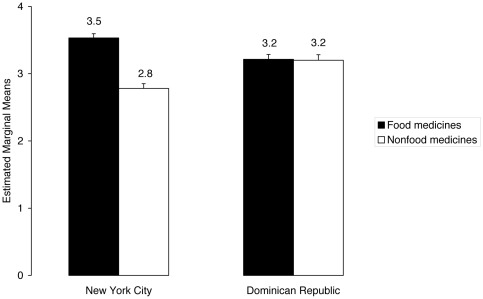
A transnational comparison of knowledge about food and nonfood medicinal plants between NYC and DR. Data is presented as square root transformation of the mean number of medicinal plants ± s.e.m. that were reported for thirty common health conditions by lay persons in NYC and DR who self-medicate with medicinal plants (N = 165 in NYC and N = 128 in DR).

A factorial MANCOVA applied to both types of plant knowledge with age as covariate (Table 1) showed that knowledge of these two types of medicinal plants (food medicines versus nonfood medicines) was affected oppositely by migration from the DR to NYC, as depicted in Figure 1. Knowledge of food medicines was higher in the group that migrated to NYC, whereas knowledge of nonfood medicines was lower. MANCOVA also showed different effects of age, place of origin (rural or urban DR), and the interaction between country and place of origin on knowledge of food and nonfood medicines (in contrast, gender had the same effect) (Table 1). People from rural areas (who grew up in the DR countryside or are currently living there) have more knowledge of nonfood medicines than people from urban areas. Both groups (rural and urban) hold the same amount of knowledge about food medicines. When migrating to NYC, a comparison of people who originated from rural areas shows that this group loses more knowledge about nonfood medicines than people from urban areas, whereas knowledge of food medicines in these two groups appears to be unaffected.

**Table 1:** MANCOVA test of between-subjects effects in relation to knowledge of food and nonfood plants used as medicines by Dominicans.

Factor	Dependent variable	F- value	P-value
Age (covariate)	Food plant knowledge	0.25	ns
	Nonfood plant knowledge	21.01	<0.0001
Country (NYC or DR)	Food plant knowledge	11.08	0.001
	Nonfood plant knowledge	15.00	<0.0001
Place of origin in the DR	Food plant knowledge	0.49	ns
(urban or rural)	Nonfood plant knowledge	36.21	<0.0001
Gender (male or female)	Food plant knowledge	25.59	<0.0001
	Nonfood plant knowledge	7.95	0.005
Country×Place of origin	Food plant knowledge	1.30	ns
	Nonfood plant knowledge	8.05	0.005
Country×Gender	Food plant knowledge	1.38	ns
	Nonfood plant knowledge	0.60	ns
Place of origin×Gender	Food plant knowledge	0.93	ns
	Nonfood plant knowledge	0.33	ns
Country×Place of origin×	Food plant knowledge	0.00	ns
Gender	Nonfood plant knowledge	0.98	ns

ns: not significant.

### Knowledge of food medicines is not affected by age, whereas younger people have less knowledge of nonfood medicines

**Figure 2:**
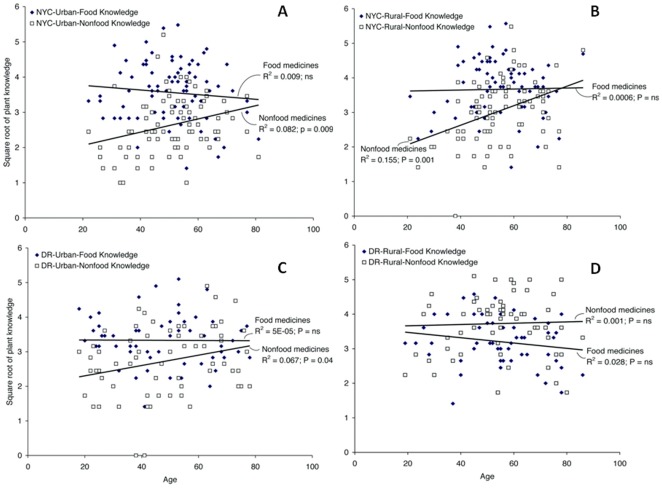
Relationship between age and knowledge of food and nonfood medicines. 2A: NYC subsample of interview participants who grew up in urban DR (N = 81); 2B: NYC subsample of interview participants who grew up in rural DR (N = 83); 2C: DR subsample of interview participants who are currently living in urban DR (N = 65); 2D: DR subsample of interview participants who are currently living in rural DR (N = 63).

There existed a general positive linear relationship between age and knowledge of nonfood medicines (r = 0.29; p<0.001). No such relationship existed for food medicines (r = 0.04; ns). The correlation between plant knowledge and age of study participants depended on people's geographic place of origin (rural or urban) in the DR. Dominicans currently living in NYC, but originating from both urban and rural DR, showed an age-dependent relationship in their knowledge of nonfood medicines (r = 0.29; p = 0.009 and r = 0.39; p = 0.001; urban and rural subsample, respectively), in contrast to knowledge of food medicines that did not vary according to age (Figures 2A and 2B). They shared this type of knowledge profile with those currently living in urban DR (r = 0.26; p = 0.04 for nonfood medicines whereas r = −0.007; ns for food medicines) (Figure 2C). However, Dominicans currently living in rural DR did not show any age-dependent effects on plant knowledge related to either food (r = −0.17; ns) or nonfood medicines (r = 0.03; ns) (Figure 2D).

## Discussion

The widely held paradigm is that plant knowledge declines with cultural change associated with modernization and globalization in many migrant and non-migrant communities world-wide [1], [2], [5], [7], [8], [11], [26], [27]. Our study demonstrated that cultural knowledge about medicinal plants in the context of a highly urbanized, transnational community in a globalized setting is kept alive and actively transformed by the geographic dynamics of that community. The increase in knowledge about food medicines in NYC was unexpected and is the first study to report a statistically measurable increase in this type of cultural knowledge associated with migration. We did not expect this to be the case in the NYC metropolis with its dramatically different ecological and social environment as compared to the DR, where people readily obtain medicinal plants free of charge from their backyards or surroundings. New Yorkers from Latino descent, however, operate a culturally-based healthcare system through *botánicas* that exists in parallel with the biomedical system [28].

Our study further demonstrated that knowledge about food plants in NYC was not age-dependent. Age-dependency of plant knowledge has been explained as a consequence of cultural erosion [29], or as the result of the process of knowledge acquisition during life, whereby older persons are expected to hold substantially more knowledge than younger people [30]. However, this study shows that younger people can possess the same amount of knowledge than older people, contradicting the hypothesis of gradual knowledge acquisition during life.

This paradigm shift has important implications for public health. Awareness about continued medicinal plant use by immigrant communities and about the dynamics in the transformation of plant use is important to inform the clinical practice, because medicinal plant use may interfere with the use of and adherence to prescribed biomedicine. Medical education, particularly as related to provision of public healthcare to minority and underserved communities, is now beginning to take into account the cultural knowledge, beliefs and practices of these communities [23], [24], [31], [32]. Ethnobotanists have an important role to play in medical education as facilitators of these cultural traditions with the goal to improve the quality of primary healthcare for minority communities through cultural competency training. Culturally competent healthcare fosters sensitivity to the cultural context of sickness and healing, including self-treatment with medicinal plants, and encourages practitioners to negotiate treatment that is acceptable to both clinician and patient [32]. This is all the more important since the United States continues to become more ethnically diverse. By 2050, Latinos or Hispanics, already the largest immigrant group in the United States, will triple in size [33].

One limitation of our study is that it focused only on first-generation immigrants, people who were born in the Dominican Republic and then moved to New York City. It is to be expected that more pronounced changes in plant knowledge will be found in subsequent generations who are born in New York City. In Micronesia, it was shown that certain cultural skills involving the use of plants, such as canoe building, were subject to higher rates of loss across generations than others, such as the planting of a cultural crop [34]. A cross-generational survey into medicinal plant use versus utilization of biomedical healthcare would be a next step to elucidate the longer term dynamics in the use of parallel healthcare systems by minority communities.

## Materials and Methods

### Study design

Institutional Review Board approval for this study was granted by the City University of New York (IRB# 04-06-0599; PI Michael J. Balick). Methods from anthropology and botany were used and consisted of surveys (interviewing participants by means of a detailed questionnaire) and botanical voucher collection followed by plant identification. The study sample consisted of 165 Dominican immigrants living in NYC who were born in the DR (105 women and 60 men), and 128 Dominicans currently living in the DR (79 women and 49 men). Psychosocial variables of these participants are listed in Table S1. For a map of the study areas see [35]. Geographic study areas in the DR of NYC and DR study participants were matched (area where they spent their childhood for the NYC subsample or area where participants were currently living for the DR subsample) (Tables S2 and S3). Most participants interviewed in NYC originated from the North-Central province of Santiago (27%), followed by the province of the capital Santo Domingo in the South (21%). Apart from these two large urban centers, two neighboring rural provinces were also chosen as study areas for interviewing in the DR: La Vega province that neighbors Santiago (10% of NYC interviewees) and the province of San Pedro de Macorís that neighbors Santo Domingo in the South-East (4% of NYC interviewees). The provinces of San Francisco de Macorís and Espaillat that precede San Pedro de Macorís in terms of number of participants are both situated in the North of the DR and therefore not selected. Interviewees were lay persons (who use medicinal plants for self-care but who do not identify themselves as plant specialists or traditional healers, or are not identified as such by others). Participants were recruited through convenience sampling, including snowball sampling. Inclusion criteria were: (a) age 18 or older; (b) born in the DR; and (c) self-reported familiarity with or some knowledge of medicinal plants. People were asked if they knew or had ever used one, two or a few medicinal plants and were assured that they did not have to be specialists in this subject so that they would feel comfortable being interviewed.

### Interviews

The questionnaires were developed and pretested before the start of the surveys. On the questionnaire, each participant received an ID number to guarantee anonymity. The DR questionnaire followed the same outline of questions and format as in NYC but was adapted to the local context. Out of respect for the fact that some interviewees may lack reading or writing skills, oral informed consent was obtained individually prior to each interview. This procedure was approved by the IRB committee. A letter that explained information about the study was read out loud to each participant and oral consent was acknowledged by checking a box and writing the date on the interview questionnaire. If the participant consented to being recorded during the interview, oral consent was also tape-recorded. The interview was conducted in Spanish, with the interviewer asking questions and recording the answers. To quantify plant knowledge (number of plants reported) for thirty common health conditions, listed in [20], and given in Table S4 the interviewers asked “Do you know of a medicinal plant to treat condition X”? After affirmation, the Spanish local name(s) of the plant, plant part used, preparation, and mode of administration were recorded. The same question was repeated for each health condition on the list. A final question gauged whether participants knew of other plants than the ones they had mentioned previously to guarantee comprehensiveness of data collection. Health conditions for the interviews were chosen based on: (1) their estimated prevalence in the Dominican community living in NYC or the DR; and/or (2) the involvement of inflammation in the pathophysiology of certain conditions (since another part of the project involved testing of crude plant extracts for anti-inflammatory activity).

### Plant identification

Because there sometimes exist different local synonyms for the same botanical species, it was essential to photograph and voucher all plants discussed. The phenomenon of parallel names for the same species is not uncommon in Dominican traditional medicine, e.g. guaucí and perriquito are two different local names for *Ruellia tuberosa* L., depending on the person interviewed and the geographic origin of that person in the DR. Following the interviews, a final list of common Spanish plant names was compiled. In order to link common names to their unique botanical identifiers (Latin names), dried and fresh reference plant samples were purchased from NYC *botánicas* (Latino healing shops that sell plant remedies imported from the Caribbean and elsewhere). In addition, voucher collections were made in NYC (for cosmopolitan species) and in the DR (for the majority of species). Plants were identified to species level with floras and other reference works [36] with assistance from plant taxonomists at The New York Botanical Garden (NYBG) and, for Dominican plants, at the Jardín Botánico Nacional “Rafael A. Moscoso” in Santo Domingo. Reference samples and voucher specimens are deposited at these institutions.

### Statistical analysis

Statistical analysis was performed with SPSS 11.5 and SigmaStat 2.0. Before analysis, data was verified for skewness, kurtosis and normality with Kolmogorov-Smirnov and Shapiro-Wilk tests. Raw data used for MANCOVA was first subjected to square root transformation to enhance normality and equality of variances. The dependent variables in MANCOVA were number of reported food medicines and nonfood medicines, respectively. Independent (fixed) variables were gender, country, and place of origin. The latter represented the geographic area (urban or rural) a participant was currently living in (for the DR sample group) or grew up in before migration (for the NYC sample group). Participant age was the covariate. Levene's test was used to verify equality of error variances in order to test the null hypothesis that the error variance of each dependent variable in MANCOVA was equal across groups. Box's test was applied to check equality of covariance matrices in MANCOVA. Correlation between age and plant knowledge was tested with Pearson product moment correlation.

## Supporting Information

Table S1 Psychosocial variables of participants in NYC and the DR.
(DOC)

Table S2 Province where NYC participants reported to have spent their childhood.
(DOC)

Table S3 Province where DR participants reported to be currently living.
(DOC)

Table S4 Overview of thirty health conditions (Spanish name or description given in brackets) used as a prop during interviews to solicit information from participants about their plant knowledge to treat these conditions.
(DOC)
