# Supplementary material for: Globalization and Loss of Plant Knowledge: Challenging the Paradigm
Source: PLoS One. 2012 May 25;7(5):e37643. doi: 10.1371/journal.pone.0037643 (PMC3360753; doi:10.1371/journal.pone.0037643)
Supplement: Table S1 — Psychosocial variables of participants in NYC and the DR. (DOC) [file pone.0037643.s001.doc]

Table S1: Psychosocial variables of participants in NYC and the DR

| **Variable** | **NYC (% of total)** | **DR (% of total)** |
| --- | --- | --- |
|  | **N=165** | **N=128** |
| Female | 105 (64%) | 79 (62%) |
| Male | 60 (36%) | 49 (38%) |
| Average age (years) | 52.9 ± 12.8 (range: 21-86) | 51.9 ± 17.0 (range: 18-86) |
|  | Median: 53 | Median: 53 |
| Rural* | 83 (50%) | 63 (49%) |
| Urban* | 81 (49%) | 65 (51%) |
| Average age when left | 34.1 ± 15.0 (range: 5-81) | N/A |
| DR (years) | Median: 32 |  |
| Average # of years in | 18.2 ± 11.0 (range 0.1-45) | N/A |
| United States | Median: 16 |  |
| Employed | 70 (42%) | 55 (43%) |
| Not employed | 95 (58%) | 73 (57%) |
| Average # of travels to | 2.9 ± 3.3 (range: 0-25) | N/A |
| DR over last 5 years | Median: 2 |  |

Numbers represent totals with proportions in brackets, unless given as average ± s.d.;*Refers to the town where they spent their childhood for NYC participants and current town for DR participants; N/A: Not Applicable.
