# Supplementary material for: Globalization and Loss of Plant Knowledge: Challenging the Paradigm
Source: PLoS One. 2012 May 25;7(5):e37643. doi: 10.1371/journal.pone.0037643 (PMC3360753; doi:10.1371/journal.pone.0037643)
Supplement: Table S2 — Province where NYC participants reported to have spent their childhood. (DOC) [file pone.0037643.s002.doc]

Table S2: Province where NYC participants reported to have spent their childhood

|  | **# of participants** | **% of total** |
| --- | --- | --- |
| Santiago | 45 | 27 |
| Distrito Nacional/Santo Domingo | 34 | 21 |
| La Vega | 16 | 10 |
| San Francisco de Macorís | 12 | 7 |
| Espaillat | 7 | 4 |
| San Pedro de Macorís | 6 | 4 |
| Salcedo | 6 | 4 |
| Puerto Plata | 5 | 3 |
| Peravia | 5 | 3 |
| Valverde | 4 | 2 |
| San Cristóbal | 4 | 2 |
| Barahona | 3 | 2 |
| Monte Cristi | 3 | 2 |
| Sanchez Ramirez | 3 | 2 |
| Azua | 2 | 1 |
| Maria Trinidad Sanchez | 2 | 1 |
| Monseñor Nouel | 2 | 1 |
| Elías Piña | 1 | 1 |
| La Romana | 1 | 1 |
| Samaná | 1 | 1 |
| San Juan | 1 | 1 |
| Santiago Rodriguez | 1 | 1 |
| **Total** | **164** | **100** |

N = 164 (missing value from one participant)
