# Supplementary material for: Globalization and Loss of Plant Knowledge: Challenging the Paradigm
Source: PLoS One. 2012 May 25;7(5):e37643. doi: 10.1371/journal.pone.0037643 (PMC3360753; doi:10.1371/journal.pone.0037643)
Supplement: Table S3 — Province where DR participants reported to be currently living. (DOC) [file pone.0037643.s003.doc]

Table S3: Province where DR participants reported to be currently living

| **Province** | | **# of participants** | | **% of total** |
| --- | --- | --- | --- | --- |
| Santiago | | 53 | 41 | |
| Distrito Nacional/Santo Domingo | | 34 | 27 | |
| San Pedro de Macorís | | 19 | 15 | |
| La Vega | | 18 | 14 | |
| Other provinces | 4 | | 3 | |
| **Total** | | **128** | **100** | |
