# Supplementary material for: Globalization and Loss of Plant Knowledge: Challenging the Paradigm
Source: PLoS One. 2012 May 25;7(5):e37643. doi: 10.1371/journal.pone.0037643 (PMC3360753; doi:10.1371/journal.pone.0037643)
Supplement: Table S4 — Overview of thirty health conditions (Spanish name or description given in brackets) used as a prop during interviews to solicit information from participants about their plant knowledge to treat these conditions. (DOC) [file pone.0037643.s004.doc]

Table S4: Overview of thirty health conditions (Spanish name or description given in brackets) used as a prop during interviews to solicit information from participants about their plant knowledge to treat these conditions

| A. diabetes (*diabetes/azúcar*) |
| --- |
| B. asthma/ chest congestion (*asma/pecho apretado*) |
| C. diarrhea (*diarrea*) |
| D. skin conditions (*problemas de la piel)* |
| 1. wounds (*heridas*) |
| 1. herpes zoster (*culebrilla*) |
| 1. skin fungi (*hongos de la piel*) |
| 1. Pityriasis (*paños*) |
| 1. furuncles (*nacíos*) |
| 1. burns (*quemaduras*) |
| E. musculoskeletal disorders (*dolores de los huesos y músculos*) |
| 1. rheumatism/arthritis (*reumatismo/artritis*) |
| 1. sprains (*torcedura/esguince*) |
| 1. trauma (*traumatismo de golpe*) |
| 1. back pain (*dolor de espalda*) |
| F. respiratory infections (*infecciones respiratorias*) |
| 1. bronchitis (*bronquitis*) |
| 1. cough (*tos*) |
| 1. flue (*gripe*) |
| 1. cold (*resfriado/catarro*) |
| 1. sinusitis (*sinusitis*) |
| G. sexual and reproductive health problems (*salud sexual y reproductiva*) |
| 1. infertility (*infertilidad/cuando alguien no puede tener bebes y quiere tener*) |
| 1. contraception (*contracepción/para ya no tener más bebes*) and abortion (*aborto/para bajar la menstruación*) |
| 1. menstrual pain (*dolor menstrual*) |
| 1. vaginal discharge (*flujo vaginal/flor blanca*) |
| 1. labor pain and puerperium (*dolor del parto y después del parto*) |
| 1. gonorrhea (*gonorrhea/enfermedad de la calle/enfermedad venérea*) |
| 1. syphilis (*sífilis/enfermedad de la calle/enfermedad venérea*) |
| 1. impotence (*impotencia/fortaleza del hombre*) |
| 1. high cholesterol (*colesterol alto*) |
| 1. blood pressure (*presión*) |
| 1. high blood pressure (*presión alta*) |
| 1. low blood pressure (*presión baja*) |
| 1. kidney problems and kidney stones (*problemas de riñones y botar piedras*) |
